# Dogs Supporting Human Health and Well-Being: A Biopsychosocial Approach

**DOI:** 10.3389/fvets.2021.630465

**Published:** 2021-03-30

**Authors:** Nancy R. Gee, Kerri E. Rodriguez, Aubrey H. Fine, Janet P. Trammell

**Affiliations:** ^1^Department of Psychiatry, Center for Human Animal Interaction, School of Medicine, Virginia Commonwealth University, Richmond, VA, United States; ^2^Human-Animal Bond in Colorado, School of Social Work, Colorado State University, Fort Collins, CO, United States; ^3^Department of Education, California State Polytechnic University, Pomona, CA, United States; ^4^Division of Social Sciences and Natural Sciences, Seaver College, Pepperdine University, Malibu, CA, United States

**Keywords:** dog, human health, human-animal interaction, biopsychosocial, canine, mental health

## Abstract

Humans have long realized that dogs can be helpful, in a number of ways, to achieving important goals. This is evident from our earliest interactions involving the shared goal of avoiding predators and acquiring food, to our more recent inclusion of dogs in a variety of contexts including therapeutic and educational settings. This paper utilizes a longstanding theoretical framework- the biopsychosocial model- to contextualize the existing research on a broad spectrum of settings and populations in which dogs have been included as an adjunct or complementary therapy to improve some aspect of human health and well-being. A wide variety of evidence is considered within key topical areas including cognition, learning disorders, neurotypical and neurodiverse populations, mental and physical health, and disabilities. A dynamic version of the biopsychosocial model is used to organize and discuss the findings, to consider how possible mechanisms of action may impact overall human health and well-being, and to frame and guide future research questions and investigations.

## Introduction – A Historical Perspective on Dog-Human Relationships

The modern relationship between humans and dogs is undoubtedly unique. With a shared evolutionary history spanning tens of thousands of years (1), dogs have filled a unique niche in our lives as man's best friend. Through the processes of domestication and natural selection, dogs have become adept at socializing with humans. For example, research suggests dogs are sensitive to our emotional states (2) as well as our social gestures (3), and they also can communicate with us using complex cues such as gaze alternation (4). In addition, dogs can form complex attachment relationships with humans that mirror that of infant-caregiver relationships (5).

In today's society, dog companionship is widely prevalent worldwide. In the United States, 63 million households have a pet dog, a majority of which consider their dog a member of their family (6). In addition to living in our homes, dogs have also become increasingly widespread in applications to assist individuals with disabilities as assistance dogs. During and following World War I, formal training of dogs as assistance animals began particularly for individuals with visual impairments in Germany and the United States (7). Following World War II, formal training for other roles, such as mobility and hearing assistance, started to increase in prevalence. Over the decades, the roles of assistance dogs have expanded to assist numerous disabilities and conditions including medical conditions such as epilepsy and diabetes and mental health disorders such as posttraumatic stress disorder (PTSD). At the same time, society has also seen increasing applications of dogs incorporated into working roles including detection, hunting, herding, and protection (8, 9).

In addition to these working roles, dogs have also been instrumental in supporting humans in other therapeutic ways. In the early 1960s, animal-assisted interventions (AAI) began to evolve with the pioneering work of Boris Levinson, Elizabeth O'Leary Corson, and Samuel Corson. Levinson, a child psychologist practicing since the 1950s, noticed a child who was nonverbal and withdrawn during therapy began interacting with his dog, Jingles, in an unplanned interaction. This experience caused Levinson to begin his pioneering work in creating the foundations for AAI as an adjunct to treatment (10). In the 1970s, Samuel Corson and Elizabeth O'Leary Corson were some of the first researchers to empirically study canine-assisted interventions. Like Levinson, they inadvertently discovered that some of their patients with psychiatric disorders were interested in the dogs and that their patients with psychiatric disorders communicated more easily with each other and the staff when in the company of the dogs (11, 12). Over the following decades, therapy dogs have been increasingly found to provide support for individuals with diverse needs in a wide array of settings (13).

## Theoretical Framework for Dog Interaction Benefits

**Figure 1:**
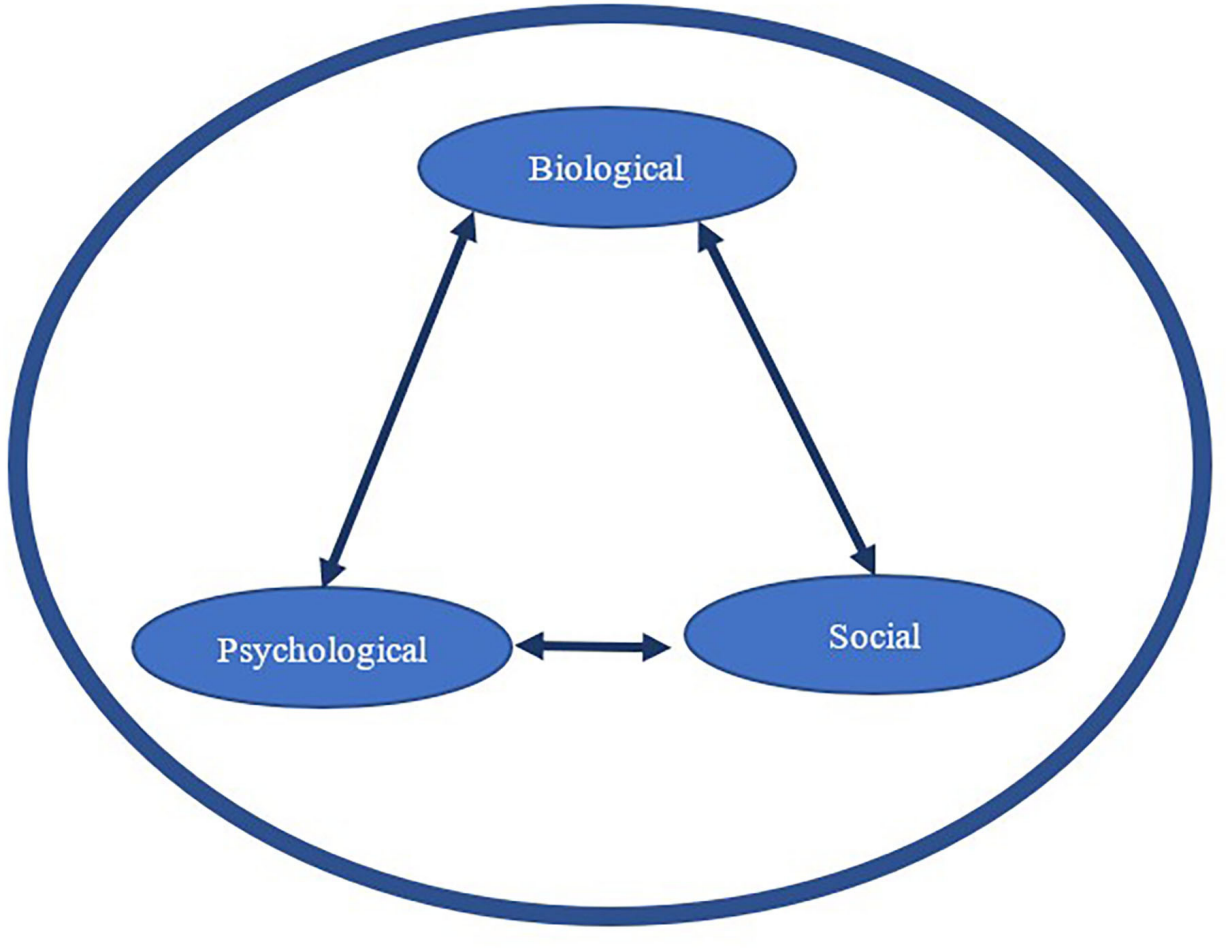
A biopsychosocial perspective of how biological, psychological, and social influences may impact one another (solid lined arrows) and influence human health and well-being (represented here by the large thick circular shape).

For over 40 years, the biopsychosocial model (14) has been widely used to conceptualize how biological, psychological, and social influences combine to determine human health and well-being. Biological influences refer to physiological changes such as blood pressure, cortisol, and heart rate, among others; psychological influences include personality, mood, and emotions, among others; and social influences refer to cultural, socio-economic, social relationships with others, family dynamics, and related matters. Figure 1 presents a graphical illustration of the relationship among these three influences in determining overall health and well-being. Although the model has dominated research and theory in health psychology for decades, more recently, it was re-envisioned as a more dynamic system (15) that construes human health as the result of the reciprocal influences of biological, psychological and social factors that unfold over personal and historical time. For example, if a person breaks his/her arm, there will be a biological impact in that immune and muscle systems respond and compensate. Social, or interpersonal, changes may occur when support or assistance is offered by others. Psychological changes will occur as a result of adjusting to and coping with the injury. Thus, the injury represents a dynamic influence initiated at one point in time and extending forward in time with diminishing impact as healing occurs.

This dynamic biopsychosocial approach to understanding health and well-being is appealing to the field of human-animal interaction (HAI) because of the dynamic nature of the relationship between humans and animals. For example, a person may acquire many dogs over his/her lifetime, perhaps from childhood to old age, and each of those dogs may sequentially develop from puppyhood to old age in that time. Behaviorally, the way the human and the dog interact is likely to be different across the lifespans of both species. From a biopsychosocial model perspective, the dynamic nature of the human-canine relationship may differentially interact with each of the three influencers (biological, psychological, and social) of human health and well-being over the trajectories of both beings. Notably, these influencers are not fixed, but rather have an interactional effect with each other over time.

While a person's biological, psychological, and social health may affect the relationship between that person and dogs with whom interactions occur, the focus of this manuscript is on the reverse: how owning or interacting with a dog may impact each of the psychological, biological, and social influencers of human health. We will also present relevant research and discuss potential mechanisms by which dogs may, or may not, contribute to human health and well-being according to the biopsychosocial model. Finally, we will emphasize how the biopsychosocial theory can be easily utilized to provide firmer theoretical foundations for future HAI research and applications to therapeutic practice and daily life.

### Psychological Influences

Much research has been conducted on the impact of dog ownership and dog interactions on human psychological health and functioning. Frequent interactions with a dog, either through ownership or through long-term interventions, have been associated with positive psychological outcomes across the lifespan [for a systematic review of this evidence see (16)]. One psychological aspect of interest to many HAI researchers is depression, especially among older adults. However, the relationship of pet dog ownership and depression over the lifespan continues to have inconsistent and inconclusive findings (16). Nevertheless, there are examples in the literature highlighting the beneficial role of dog ownership in reducing depression. As is frequently the case in HAI, the evidence from intervention studies is stronger than that of pet ownership studies (16), with the preponderance of this evidence linking animal-assisted interventions to a decrease in depression, as measured by self-report indices. Among the mechanisms for this reduction in depression are biological and social influences. For example, one such study found that an attachment relationship with a pet dog may serve as a coping resource for older women by buffering the relationship between loneliness (also measured by self-report indices) and depression, such that the presence of the pet dog appears to ameliorate the potential for loneliness to exacerbate depression (17). A causal relationship between dog ownership and mental health is difficult to determine. Not only may owning a pet dog increase stress, but those who are already suffering from loneliness or depression may be more inclined to have a pet dog than those who do not.

Another psychological outcome related to dog interaction that receives considerable research attention is anxiety. Studies have found that short-term, unstructured interactions with a therapy dog can significantly reduce self-reported anxiety and distress levels [e.g., (18)]. For example, children with their pet dog or a therapy dog present during a stressful task exhibit lower perceived stress and more positive affect compared to when alone (19), when a parent was present (20), or when a stuffed dog was present (21). In addition to psychological mechanisms, there are social and biological mechanisms at play as well. In these short-term stressful contexts, a dog may serve as both a comforting, nonjudgmental presence as well as a positive tactile and sensory distraction. Dog interaction might also reduce anxiety and distress by influencing emotion regulation while coping with a stressor (22). During animal-assisted therapy, having a dog present during psychotherapy such as cognitive behavioral therapy can aid in decreasing self-reported anxious arousal and distress for patients who have experienced trauma, making the therapeutic treatment process more effective (23).

In addition to the negative aspects of psychological functioning, HAI research has also aimed to quantify the effects of dog interaction and ownership on positive psychological experiences such as happiness and well-being. Some studies have found that dog ownership is associated with higher life satisfaction and greater well-being (24), while other studies show that this is the case only when the dog provided social support (25) or satisfied the owner's needs (26). However, other large-scale surveys have found no significant differences in self-reported happiness between dog owners, cat owners, and non-pet owners (27), contributing to mixed findings. Recent discussions argue that too much focus has been placed on the relationship between mental health and the simple variable of dog ownership, when the specific activities that owners engage in with their dogs (e.g., walking, tactile interaction, and shared activities,) may be more important in explaining positive well-being (28). Further, many other factors may be driving these inconsistent findings in depression, anxiety, and well-being, including the owner's personality (24), gender and marital status (29), and attachment to the dog (30).

Dogs may also provide a source of motivation; for example, people with dogs are more likely to comply with the rigors of their daily life (31). The relationship with a pet dog may provide motivation to do things that may be less desirable. For example, for older adults who own pets, it is not uncommon for them to be more involved in daily life activities because of the need to take care of their animals (32). Likewise, children also complete less desired activities due to their relationship with the dog [for a discussion of this topic see (33)].

An accumulation of research also suggests that dog interaction may have specific psychological benefits for individuals with physical disabilities and chronic conditions. Cohabitating with a specially trained assistance dog, including guide, hearing, and service dogs, can be associated with increased psychological and emotional functioning among individuals with disabilities (34). For individuals with mental disorders such as posttraumatic stress disorder (PTSD), recent research has also found that having a psychiatric service dog is associated with fewer PTSD symptoms, less depression and anxiety, and better quality of life [For a review see (35)]. These benefits appear to be due to a combination of the service dog's specific trained tasks and aspects inherent to cohabitating with a pet dog, including having a source of love, nonjudgmental social support, and companionship (36).

Similar research has also highlighted the value of dogs for children with disorders of executive functioning and self-regulation, especially autism spectrum disorder (ASD) and attention-deficit/hyperactivity disorder (ADHD). For some children with ASD, dogs may provide a calming and positive presence (37) and may both reduce anxiety (38) and improve problematic behaviors (39). Parents report that both pet dogs and service dogs can provide certain benefits for children with ASD, including benefits to children's moods, sleep, and behavior (40, 41). Therapy dogs have also been found to be impactful in supporting children with ADHD in their emotional regulation (42) and aspects of character development (43). Nevertheless, the outcome of dog interactions may not be positive for all individuals with ASD and ADHD; despite evidence of psychological benefits of dog interaction for some children, others may be fearful or become over-stimulated by dogs (44).

In addition to impacts on psychological health, dog interaction can also impact psychological functioning, cognition, and learning. Among children, emerging research suggests short-term interactions with a therapy dog may lead to improvements in specific aspects of learning and cognition. A recent systematic review of research on therapy dog reading programs indicated that reading to a dog has a number of beneficial effects including improved reading performance (45). Studies suggest that interacting with a therapy dog may also improve speed and accuracy on cognitive (e.g., memory, categorization, adherence to instructions) and motor skills tasks among preschool-aged children compared to interacting with a stuffed dog or human (46). Similarly, a recent study showed that 10–14-year-old children had greater frontal lobe activity in the presence of a real dog as compared to a robotic dog, indicating a higher level of neuropsychological attention (47).

Among young adults, similar effects on cognition and learning have been found. Numerous colleges and universities now offer interactions with therapy dogs, typically during high stress times (such as before exams). In this sense, a biological mechanism through which dog interaction may positively impact cognition and learning is via stress reduction and improvement in positive affect. Even such short and infrequent interactions with therapy dogs may decrease perceived stress and increase perceived happiness in college students [e.g., (48, 49)]. Further, some institutions have permanent resident therapy dogs and/or long-term intervention programs; one such program showed that students who interacted with therapy dogs for 8 weeks reported significantly less homesickness and greater satisfaction with life than wait-listed controls (50). These effects may translate to additional effects on students' academic success, learning, and cognition. For instance, a recent randomized controlled trial (51) paired a standard academic stress management program with therapy dog interaction; the pairing produced significantly higher levels of self-reported enjoyment, usefulness, self-regulation, and behavior change than the stress management program or dog interaction alone. However, when therapy dog interaction is closely paired with more specific learning experiences, beneficial effects on stress remain, but benefits to academic performance may not manifest. For example, a recent study showed that interacting with a therapy dog resulted in significant improvements in students' perceived stress and mood, but not in actual exam scores (52). Similarly, interacting with a therapy dog during the learning and recall phase of a memory test did not improve memory compared to a control group (53). Taken together, dog interaction may improve stress and affect among college-aged adults as well as dimensions important for academic success and learning, but these results may or may not translate to cognitive performance benefits.

### Biological Influences

The psychological and biological effects of HAI are often closely interwoven, as seen in the Psychological Influences section above and as demonstrated by the frequency with which psychological effects are evaluated using biological assessments of stress, anxiety, and arousal (54). For example, a plethora of studies have examined how short-term interactions with dogs can influence stress by measuring physiological biomarkers. Studies have found that dog interaction can influence parameters such as blood pressure, heart rate, and electrodermal activity (55) as well as neurochemical indicators of affiliative behavior [e.g., beta-endorphins, prolactin, and dopamine; (56)].

However, one of the most popular physiological measures in HAI research is the stress hormone cortisol (57). Studies have found that short-term interactions with a dog can decrease both subjective stress and circulating cortisol concentrations [e.g., (58)]. Cohabitating with a dog has also been found to impact circulating cortisol after waking among children with ASD (39) and military veterans with PTSD (59). Experimental studies have also examined how having a dog present may modulate the stress response and cortisol secretion among individuals undergoing a stressful situation. Among adults, studies have found that having a dog present during a socially stressful paradigm can attenuate cortisol compared to when alone or with a human friend (60). A recent randomized controlled trial similarly found that interacting with a therapy dog, for 20 min, two times per week, over a 4-week period resulted in reduced cortisol (basal and diurnal measurement) among typically developing and special needs school children compared to the same duration and length of delivery for a yoga relaxation or a classroom as usual control group (61). However, it is of note that many methodologically rigorous studies have not found significant effects of interacting with a dog on physiological parameters, including salivary cortisol (21, 62, 63). A recent review of salivary bioscience research in human-animal interaction concluded that significant variation exists with regards to sampling paradigms, storage and assaying methods, and analytic strategies, contributing to variation in findings across the field (57).

As research quantifying the physiological outcomes from dog interaction continues to increase, so does research attempting to understand the underlying mechanisms of action leading to stress reduction. One theoretical rationale for dogs' stress-reducing benefits consists of the dog's ability to provide non-judgmental social support (60), improve positive affect (64), and provide a calming presence (22). Dogs may also contribute to a feeling of perceived safety and provide a tactile and grounding comfort (65). For these reasons, dogs are often incorporated into treatment and recovery for individuals who have experienced a traumatic event (66). Another mechanism contributing to these stress reducing benefits may be tactile stimulation and distraction derived from petting or stroking a dog. For example, Beetz et al. (67) found that the more time a child spent stroking the dog before a stressful task, the larger the magnitude of cortisol decrease. In fact, calming tactile interactions such as stroking, touching, and petting may be a key mechanism explaining animal-specific benefits to stress physiology, as touch is more socially appropriate in interactions with animals than as with other people (22). While there are many hypothesized mechanisms underlying positive psychophysiological change following human-dog interaction, more research is needed to determine how individual differences in humans, animals, and the human-animal relationship affects outcomes (21, 57, 62, 63).

Another mechanism in which positive dog interaction may result in psychophysiological benefits is via the secretion of oxytocin. Oxytocin not only buffers the stress response and cortisol secretion (68) but is also involved emotion, trust, and bonding (69). The oxytocin system has been hypothesized to be a primary mechanistic pathway involved in human-dog interactions (70). Positive dog-owner interactions including stroking, petting, and talking have been shown to result in increased oxytocin levels in both dog owners and dogs, which has been related to the strength of the owner-dog relationship (71) and dog-human affiliative behaviors (72, 73). Some studies have also found differential effects in oxytocin reactivity after dog interaction between human males and females (74), giving context to potential gender and/or hormonal differences in dog-human interactions. However, even though the oxytocin system exhibits potential as a pathway by which dogs provide psychophysiological benefits, it should be noted that mixed findings and methodological and measurement differences limit strong conclusions (75).

In regards to pet dog ownership, many studies have also sought to understand the biological effects of long-term interactions with a pet dog. Some research suggests that sharing animal-associated microbes with a pet dog can have long-term impacts on human health (76) while others have found that cohabitating with a pet dog can be beneficial for child allergies (77) and immune system development (78). However, most research on the long-term health impacts of pet dog ownership has focused on cardiovascular functioning. Epidemiological research suggests that dog ownership is linked to greater physical activity levels (presumably linked to dog-walking), and reduced risk for cardiovascular disease, stroke, and all-cause mortality [for a summary see (79)]. A recent meta-analysis of ten studies amassing data from over three million participants found that pet dog ownership was associated with a 31% risk reduction for mortality due to cardiovascular disease (80). However, dog ownership research of this nature will always suffer from an important chicken and egg type question: do dogs make us healthier, or do healthy people opt to own dogs?

### Social Influences

A final way in which dog companionship and interaction may contribute to human health and well-being is through the social realm. Dogs may impact social functioning by providing direct social support (81) and a source of an attachment bond (82) which in turn may contribute to better social and mental health by providing companionship. Acquiring a pet dog has been reported to reduce both short-term and long-term self-reported loneliness (83). Particularly for those who live alone, dog ownership may serve as a protective factor against loneliness in times of social isolation, such as during the COVID-19 pandemic (84). Among older adults living in long-term care facilities or who live alone, dog visitation may also decrease loneliness by providing a source of meaningful companionship and social connectedness (85, 86). However, the literature on pet dogs and loneliness is also characterized by mixed findings, raising the possibility that dog ownership may be a *response* to loneliness rather than *protection* from loneliness. Further, there remains a lack of high quality research in this area which limits any causal conclusions (87).

Another way in which the social support from a pet dog may benefit social functioning is by facilitating social interactions with others. For example, observational studies have found that being accompanied by a dog in public increases the frequency of received social interactions (88) and social acknowledgments [e.g., friendly glances, smiles; (89)]. For those who engage in dog walking, social interactions are perceived as a rewarding side effect (90). Dogs can also provide a source of social capital, defined as the glue that holds society together (91). The research of Wood and colleagues (92) suggests that dogs can function as facilitators for social contact and interaction, with pet owners reporting higher perceptions of suburb friendliness and more social interactions with neighbors compared to non-pet owners.

For children and adolescents, pet dog ownership may contribute to healthy social development. Positive child–pet dog interactions have been shown to have benefits to children's social competence, interactions, and play behavior [for a review see (93)]. Not only can children form attachment relationships with dogs (94), but pet dogs may promote feelings of safety and security (95) that can facilitate childhood social development. Pet ownership may also help children develop skills to form and maintain social relationships with their peers (96). For example, cross-sectional studies found that children with a pet dog in the home have fewer peer problems and have more prosocial behavior with children without a dog [e.g., (97, 98)].

Among children with developmental disorders, dog interaction has also been similarly shown to impact social functioning. For children with ADHD, two randomized controlled trials have found that 12 weeks of visits with a therapy dog, incorporated into curricula designed to improve skills and reduce behavioral problems, can result in improved social skills, prosocial behaviors, and perceptions of social competence (42, 43). One potential explanation for these benefits is that children may interpret the dogs' nonverbal communication as less threatening and easier to interpret than human interaction (99, 100). A recent eye-tracking study found that children with ASD exhibit a bias in social attention to animal faces, including dogs, compared to human faces (101). The presence of a dog in clinical applications may also promote more social engagement with a therapist while reducing negative behaviors (102, 103). Further, there is some evidence that having a pet dog in the home can have a positive impact on social interactions of children with ASD, especially among verbal children, while teaching children responsibility and empathetic behavior (104, 105).

## Potential Mechanisms of Action

We have discussed how, in the psychological realm, interacting with a dog can positively relate to depression, anxiety, and well-being as well as psychological functioning in the areas of cognition, learning, and attention. It is interesting to note that most psychological constructs are measured using self-report indices, such as the Beck Depression Inventory (106) or the UCLA Loneliness Scale (107), while a smaller group of constructs are measured using speed and accuracy to detect targets (attention) or to remember information (learning and memory). In the biological realm, we discussed how interacting with dogs can influence stress-related physiological parameters and long-term biological and cardiovascular health. Biological measures are often recorded in real-time, such as heart rate or blood pressure, or are collected at critical time points during the study (e.g., saliva, urine, or blood samples for such measures as cortisol or oxytocin). Finally, we discussed the social realm, in which interacting with a dog can provide social support, facilitate social interactions, and improve social development and social skills. Measures used to assess variables in the social realm include self-report indices (e.g., demographics such as marital status, numbers of family members and friends), real time observations of social interactions (e.g., video analyses of interactions using ethograms), and parent/teacher reports of social functioning [e.g., Social Skills Rating System; (108)]. To better understand and organize these various findings, we now consider potential mechanisms of action in the context of the biopsychosocial model, and as part of this discussion we will consider the potential for different types of measurement to have their own influence.

The mechanisms that underly positive human-dog interactions are likely to be interrelated and broadly, yet differentially, impactful across the three influencers of health (biological, psychological and social). According to the biopsychosocial model, impacts on one of the influencers of health is likely to impact the others (14). Further, an underlying mechanism of change may have a larger immediate impact on one realm than on the other two (15). Although this applies to the many influences we have discussed above, we will describe a reduction in stress as a more detailed example of how the biopsychosocial model can be considered. Stress is likely to have an immediate and measurable impact on the biological system through endocrinological (e.g., changes in cortisol) and psychophysiological (e.g., changes in blood pressure) processes. This same reduction in stress is likely to impact the psychological system through changes in mood or affect, concentration, and motivation, but that impact may not be immediately measurable or may be smaller in magnitude. This conjectured delay or reduction in effect size stems at least in part, from the way these changes are typically measured and the time course for potential effects to become measurable. For example, some biological changes indicative of increased stress (e.g., heart rate) can be measured in direct correspondence with the experimental manipulations (e.g., interacting with the dog vs. experiencing a control condition), and provide real time biological indications of changes in stress levels. Psychological indications of stress may be measured by a self-report survey instrument assessing state or trait anxiety. This type of measure cannot be completed in real time during the various experimental conditions (e.g., interacting with the dog vs. experiencing a control condition), but must be completed at some point following the experimental manipulation. It is possible that psychological measures are not as immediately sensitive to changes in the constructs they measure because of the required delay between manipulation and measurement. Such a delay may underestimate the real time effect as it may fade over time. Finally, reductions in stress have the potential to impact social systems by increasing social approaches and acceptance of approaches by others, but that impact may be of a small size or require even more time to be measurable. For example, exposure to stress may have immediate physiological effects, but it could take more time (prolonged exposure to stress) for those effects to impact some measures of social influence such as number of friends.

**Figure 2:**
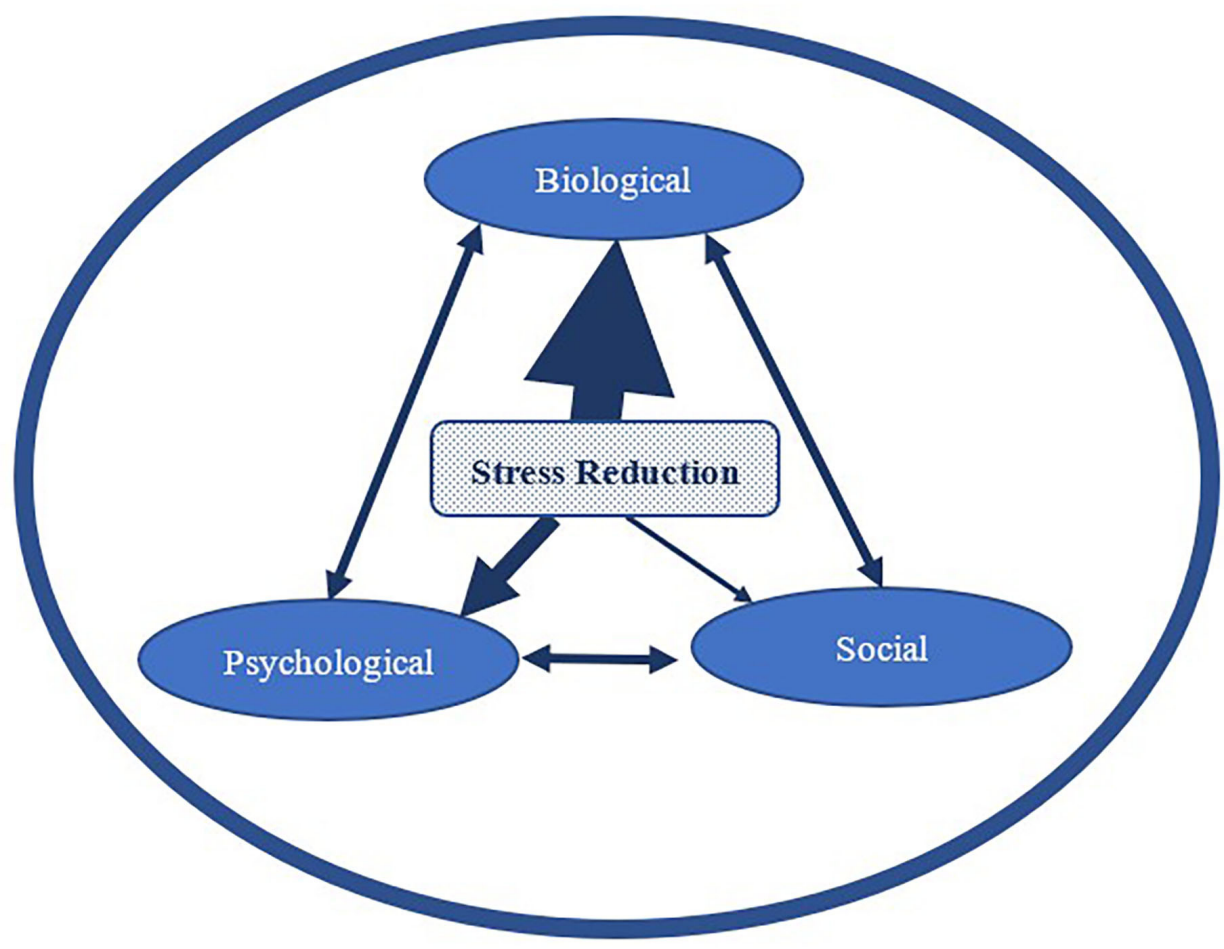
An example of the potential for differential impact (represented by the different arrow thickness) of one mechanism of action (stress reduction) on the three realms of influence of overall health and well-being (depicted by the larger encompassing circle).

In Figure 2, the mechanism of stress reduction is used as one example for the purposes of this discussion to exemplify how human-dog interactions may influence human health and well-being, as explained by the biopsychosocial model. Stress reduction may have a more immediate or larger impact on the biological realm as demonstrated by the larger arrow, while having a smaller (or perhaps delayed) impact on the psychological realm and an even smaller (or potentially more delayed) impact on the social realm.

Based on the research described earlier, we have seen that interacting with a dog can have stress reducing impacts in the biological realm such as decreased cortisol, heart rate, and blood pressure, and increases in oxytocin. In the psychological realm, stress reduction can be a driver of immediate improvements in self-report measures of stress, mood, and anxiety and more delayed improvements in overall mental health and quality of life. The social realm is also likely to be directly and indirectly impacted by this stress reduction from both immediate and delayed psychophysiological changes as well as more long-term improvements in social support, social networks, social development, and overall social health. Therefore, it is important to consider the dynamic nature of these three realms in that there may be a strong immediate effect of dog interaction on one realm, but a lesser, delayed impact in the other two realms. Similar to our more detailed example of stress above, other influences we have discussed (e.g., social support, positive affect, etc.) are likewise mechanisms that operate in a similar reciprocal biopsychosocial framework. Further, although it likely that the three influences are interrelated, it is not known from the current evidence the degree to which they may be interrelated and thus have shared and overlapping effects on one another and on overall health and well-being. Therefore, a consideration of mechanisms that influence human-dog interactions from a dynamic and flexible biopsychosocial perspective, instead of from a single realm, is an important addition to the study of human-animal interaction.

## Conclusion and Future Directions

In conclusion, the biopsychosocial model is a promising theoretical model to be applied to human-animal interaction research for several reasons. First, the field of HAI has been plagued by mixed findings in which some research suggests that dogs have beneficial effects on human health and well-being and others suggest no effect or even a negative effect [for a discussion see (109)]. This variability in HAI research outcomes caused by differing methodologies, measurement, populations, and interventions is described in detail by Rodriguez et al. (110). However, we also argue that some of the variability seen in HAI research may be explained by the potential for differential immediate and delayed impacts within each of the three biopsychosocial model realms. For example, if dog interaction shows immediate reduction in physiological measures of stress, how long does that reduction last, and do we see corresponding immediate and/or delayed responses in the psychological and social realms? Therefore, more information about differential impacts of dog interactions on each of the three influencers at various points in time is needed. In addition, it may be necessary to apply a variety of measures (at least one measure per influencer realm) over time to fully disentangle the existing mixed results in the field of HAI.

Secondly, due to the flexibility that this dynamic biopsychosocial model offers in explaining HAI research outcomes, we propose this model as an effective avenue to promote future theoretically grounded research in our field. Saleh (111) stresses that practice, research, and theory are the corner stones of any field, HAI is not exempt from this consideration. The field of HAI will benefit from applying an accepted model, like the biopsychosocial model, because it provides a useful framework for understanding and predicting how interactions between humans and animals impacts human health and well-being. As Saleh (111) explains, “it is the result of the relationship between the process of inquiry (research) and the product of knowledge (theory)” that our understanding of a process may become clearer. Therefore, current research should continue to modify and impact a present theory, which should act as a guide for researchers to constantly generate and test the basis of a theory (111). The findings from such theory-driven research could then help practitioners, as well as health care policy makers, in how to effectively incorporate dogs in therapeutic settings and in homes.

Lastly, the reciprocal relationship of the psychological, biological, and social domains can be used to elucidate the mechanisms that both impact and are impacted by interactions between humans and animals. Theory-driven science (for which we have proposed the biopsychosocial model as a useful framework) should be used to influence and inform research, practice, and policy. Thus, researchers and practitioners applying the biopsychosocial model will be instrumental not only in guiding future research in the field, but also in clarifying existing research as well people's perceptions of benefits derived from canine-human interactions.

## Author Contributions

NG provided the initial organization and theoretical framework. All authors wrote and edited the document in shared collaboration and discussed and conceived the idea for the paper.

## Conflict of Interest

The authors declare that the research was conducted in the absence of any commercial or financial relationships that could be construed as a potential conflict of interest.
